# Associated Bacteria Affect Sexual Reproduction by Altering Gene Expression and Metabolic Processes in a Biofilm Inhabiting Diatom

**DOI:** 10.3389/fmicb.2019.01790

**Published:** 2019-08-02

**Authors:** Emilio Cirri, Sam De Decker, Gust Bilcke, Markus Werner, Cristina Maria Osuna-Cruz, Lieven De Veylder, Klaas Vandepoele, Oliver Werz, Wim Vyverman, Georg Pohnert

**Affiliations:** ^1^Institute of Inorganic and Analytical Chemistry, Friedrich Schiller University Jena, Jena, Germany; ^2^Protistology and Aquatic Ecology, Department of Biology, Ghent University, Ghent, Belgium; ^3^Department of Plant Biotechnology and Bioinformatics, Ghent University, Ghent, Belgium; ^4^VIB Center for Plant Systems Biology, Ghent, Belgium; ^5^Department of Pharmaceutical/Medicinal Chemistry, Institute of Pharmacy, Friedrich-Schiller-University Jena, Jena, Germany

**Keywords:** pheromones, diatoms, bacterial exudates, cross-kingdom interactions, metabolomics, transcriptomics

## Abstract

Diatoms are unicellular algae with a fundamental role in global biogeochemical cycles as major primary producers at the base of aquatic food webs. In recent years, chemical communication between diatoms and associated bacteria has emerged as a key factor in diatom ecology, spurred by conceptual and technological advancements to study the mechanisms underlying these interactions. Here, we use a combination of physiological, transcriptomic, and metabolomic approaches to study the influence of naturally co-existing bacteria, *Maribacter* sp. and *Roseovarius* sp., on the sexual reproduction of the biofilm inhabiting marine pennate diatom *Seminavis robusta*. While *Maribacter* sp. severely reduces the reproductive success of *S. robusta* cultures, *Roseovarius* sp. slightly enhances it. Contrary to our expectation, we demonstrate that the effect of the bacterial exudates is not caused by altered cell-cycle regulation prior to the switch to meiosis. Instead, *Maribacter* sp. exudates cause a reduced production of diproline, the sexual attraction pheromone of *S. robusta*. Transcriptomic analyses show that this is likely an indirect consequence of altered intracellular metabolic fluxes in the diatom, especially those related to amino acid biosynthesis, oxidative stress response, and biosynthesis of defense molecules. This study provides the first insights into the influence of bacteria on diatom sexual reproduction and adds a new dimension to the complexity of a still understudied phenomenon in natural diatom populations.

## Introduction

Diatoms are among the most productive and ecologically relevant unicellular algae on Earth. Their high genetic diversity and adaptive potential allowed them to diversify into hundreds of genera and over 100,000 species, occurring in freshwater, marine, and soil habitats globally (Malviya et al., 2016). Moreover, they are a fundamental link in global biogeochemical cycles, contributing up to 20% of the total primary production on Earth (Field et al., 1998) and being key players in oceanic silica cycling. While planktonic diatoms have been extensively studied, benthic diatoms often also dominate primary production in biofilms in the photic zone and play an important role in regulating nutrient fluxes in and out of sediments (Smith and Underwood, 1998).

In recent years, it has become increasingly clear that diatoms engage in multiple interactions with bacteria (Amin et al., 2012; Seymour et al., 2017). Many of these are confined to the so-called phycosphere (Bell and Mitchell, 1972), a zone surrounding the microalgal cell where diffusion controls transport of exuded chemicals (Seymour et al., 2017). While some bacteria promote the growth of diatoms or show mutualistic behavior (Seymour et al., 2017), for example by releasing nutrients (Helliwell et al., 2014) or growth hormones (Amin et al., 2015), other microbes suppress diatom growth (Meyer et al., 2017) by the production of algicidal compounds (Wang and Seyedsayamdost, 2017) or growth inhibiting factors (van Tol et al., 2017; Stock et al., 2019). Therefore, diatom–bacteria interactions control nutrient cycling at the base of the foodweb and act as regulators of algal blooms (Riemann et al., 2000; Seymour et al., 2017). Because of this, studying these interactions is fundamental for understanding the ecological importance of diatoms in biogeochemical cycles, as well as their evolutionary history (Azam and Malfatti, 2007; Ramanan et al., 2016). Despite the relevance of diatom–bacteria relationships, studies to unravel the underlying molecular mechanisms remain scarce (Durham et al., 2017).

Recently, it was shown that some bacteria are able to interfere with sexual reproduction of the benthic diatom *Seminavis robusta* (Cirri et al., 2018), a motile pennate diatom inhabiting coastal biofilm communities. *S. robusta* has a heterothallic mating system in which, once the cell size drops below the sexual size threshold (SST), both mating types (MT^+^ and MT^–^) release different sex inducing pheromones (SIP^+^ and SIP^–^, respectively). These SIPs induce a temporary arrest in the cell cycle of the opposite mating type in G1 phase to synchronize the switch to gametogenesis (Frenkel et al., 2014a; Moeys et al., 2016). Moreover, SIP^+^ induces the production of an attraction pheromone by MT^–^ cells: a diketopiperazine consisting of two proline molecules called diproline (Gillard et al., 2013). This pheromone then attracts the MT^+^ cells, resulting in physical pairing of compatible cells and subsequent gametogenesis. Although diproline is stable in artificial seawater, in non-axenic cultures its concentration oscillates on a daily basis (Gillard et al., 2013; Frenkel et al., 2014b). It was recently shown that two bacteria associated with *S. robusta* (*Maribacter* sp. and *Roseovarius* sp.) are able to modulate extracellular diproline concentrations and that the exudates of both bacteria have different effects on the reproductive success of *S. robusta* (Cirri et al., 2018). Exudates of *Maribacter* sp. negatively affect the sexual reproduction of *S. robusta*, while *Roseovarius* sp. exudates slightly enhance it. Both bacterial isolates are able to degrade diproline, but only when severely nutrient-deprived. Experimental results suggest that bacterial metabolites interfere in a direct manner with the physiology of diatoms and attraction pheromone production, thereby influencing the reproductive success of *S. robusta*.

Here we combined physiological, metabolomic, and transcriptomic approaches to gain mechanistic insights into the effect of *Roseovarius* sp. and *Maribacter* sp. exudates on *S. robusta* and its sexual behavior. We analyzed the effect of both bacteria on the induced cell cycle arrest caused by SIP^+^, gene expression, and metabolic profiles in MT^–^ cells. We show that neither of the bacterial exudates affect cell cycle arrest but they both trigger an oxidative stress response in the diatom. Moreover, we show that *Maribacter* sp. affects the metabolism of several amino- and fatty acids and thereby indirectly influences diproline production. *Roseovarius* sp. enhances the expression of enzymes that synthetize precursors of the attraction pheromone.

## Materials and Methods

### Strains and Culture Conditions

*Seminavis robusta* strains 85A (MT^+^) (BCCM: DCG0105) and 84A (MT^–^) (BCCM: DCG0104) were obtained from the diatom culture collection of the Belgian Coordinated Collection of Micro-organisms (BCCM/DCG^1^). Cultures of both mating types were grown separately under a 12 h:12 h dark/light regime (cool white light at an intensity of 50 μmol m^–2^ s^–1^) at 18°C in Guillard’s F/2 medium (Guillard, 1975). This medium was prepared by autoclaving 34.5 g/L Tropic Marin^®^ BIO-ACTIF sea salt (Tropic Marin^®^, Wartenberg, Germany) and supplementing it with 20 mL/L Guillard’s (F/2) Marine Water Enrichment Solution (Sigma–Aldrich). Axenic cultures were prepared following the protocol of Cirri et al. (2018).

Stock cultures of *Roseovarius* sp. and *Maribacter* sp. isolated from *S. robusta* (for the method, see Cirri et al., 2018) were grown in Difco^TM^ Marine Broth medium at room temperature for 3 days before the experiment. Then 25 mL of the bacterial culture was transferred to a 50 mL Falcon tube, centrifuged for 3 min at 6,000 × *g*, washed three times with minimal medium (F/2 medium with 5 g/L glucose, 5 mL/L glycerol, and 1.5 g/L NH_4_NO_3_), and transferred to 500 mL of minimal medium. The cultures were grown for 10 days at room temperature until they reached the late exponential phase (OD_600_ = 0.1 measured with a Shimadzu^®^ UV-1601 Spectrophotometer) before being sterile-filtered to harvest sterile bacterial exudates.

### Harvesting of MT^+^ Medium

*Seminavis robusta* strain 85A (MT^+^) was grown at 18°C in CELLSTAR^®^ Standard Cell Culture Flasks with a 175 cm^2^ surface area, filled with 200 mL Guillard’s F/2 medium under 12 h:12 h dark:light regime (50 μmol m^–2^ s^–1^ photons of cool white light). As a proxy for the biomass in the flasks, we measured the minimum fluorescence value (*F*_0_) after 15 min of dark-adaptation. Pulse-amplitude-modulation (PAM) fluorimetry measurements were performed using a MAXI Imaging PAM Fluorimeter, M-series (Walz, Effeltrich, Germany), equipped with an IMAG-K4 camera and mounted with an IMAG-MAX/F filter. *F*_0_ was measured using the following software settings: intensity 7, gain 3, and damping 2. When the culture reached an *F*_0_-value of ≈0.35, the medium was harvested, sterile-filtered using GF/F filters (ø 47 mm) on Nalgene^TM^ reusable bottle top filters units (Thermo Fisher Scientific, Bremen, Germany) connected to sterile 250 mL Duran^®^ bottles (Schott, Jena, Germany), aliquoted in 50 mL Falcon tubes, and stored at −20°C until usage. In total, 12 culture flasks (2,4-L SIP^+^-containing medium) were harvested.

### Induction of Sexuality and Co-cultivation of *S. robusta* With Bacteria

*Seminavis robusta* strain 84A (MT^–^) was grown at 18°C in CELLSTAR^§^ Standard Cell Culture Flasks with a 175 cm^2^ surface area, filled with 200 mL Guillard’s F/2 medium under 12 h:12 h dark:light regime (50 μmol m^–2^ s^–1^ photons of cool white light). Once the cultures reached an *F*_0_-value of ≈0.30, the culture medium was renewed and the flasks were placed in complete darkness at 18°C for 24 h to synchronize the cell cycle in G1-phase (Moeys et al., 2016). After 21 h of darkness, sexuality was induced in MT^–^ cultures by removing 20 mL medium and replacing it with 20 mL SIP^+^-containing medium to end up with a final dilution of 1:10 SIP^+^. Also, after 21 h of darkness, bacterial exudates were added to the flasks, diluted to a volume equivalent to the volume of a full bacterial culture at OD_600_ = 0.05, the cell density at which the effects on sexual reproduction of these bacteria were shown (Cirri et al., 2018). Addition of SIP^+^ and/or bacterial exudates was done in a dark room to prevent progression through the cell cycle. Control cultures, where no SIP^+^ or bacterial exudates were added, were also moved to the dark room and back to avoid any differences in light treatment between control and treatment cultures. After addition of SIP^+^ and/or bacterial exudates, the cultures were placed in complete darkness at 18°C for another 3 h before the light was switched on (50 μmol m^–2^ s^–1^ photons).

All six treatments (control, SIP^+^-treated, *Roseovarius* sp.-treated, *Maribacter* sp.-treated, SIP^+^ + *Roseovarius* sp.-treated, and SIP^+^ + *Maribacter* sp.-treated) were cultured and harvested in five replicates.

### Cell Harvesting

After 10 h of light, 150 mL of the medium was poured over a GF/C filter (ø 47 mm) at 650 mbar on Nalgene^TM^ reusable bottle top filters units (Thermo Fisher Scientific, Bremen, Germany) connected to sterile 250 mL Duran^®^ bottles (Schott, Jena, Germany) without disturbing the cells. The filtrate was used for exometabolome extraction. The cells were then scraped from the surface of the culture flasks using a cell scraper and homogenized in the remaining medium (50 mL) by shaking. Ten milliliters of the cell suspension was used for flow cytometry analysis, while the remaining 40 mL of the suspension was used for RNA extraction.

### Cell Cycle Analysis Using Flow Cytometry

Of each harvested culture, 10 mL was isolated in a 15 mL falcon tube. The samples were centrifuged for 5 min at 2,000 rcf. The supernatant was discarded and the cells were fixed by resuspending the pellet in 10 mL ice cold 75% ethanol. Samples were stored in the dark at 4°C until analysis.

Fixed cultures were centrifuged for 5 min at 3,000 rpm, after which the supernatant was replaced with 2 mL ice cold 75% ethanol. One milliliter of each sample was transferred to a 1.5-mL tube and washed three times with phosphate-buffered saline (PBS) buffer to remove all remaining ethanol. The fixed cells were treated with 1 μg/mL RNAse A for 20 min at 37°C and afterward stained with SYBR green (1× concentration, Life Technologies) in the dark for 20 min.

Samples were filtered through a cell strainer with pore size of 70 μm before feeding into the flow cytometer. Flow cytometry was carried out on a Bio-Rad S3e Cell Sorter (Bio-Rad Laboratories, Inc., Hercules, CA, United States), collecting 10,000 measurements for each sample and gating was carried out in the FSC and SSC channel to remove debris signals. An unstained control sample was run first to localize the cell population. After running the samples, G1 and G2 peaks were visually selected using ProSort 1.5 (Bio-Rad Laboratories, Inc., Hercules, CA, United States).

Data analysis was carried out in R (v3.4.3^2^, R Development Core Team., 2008). Since the response variable is binary [i.e., the response of a cell is either “failure” (G1) or “success” (G2-M)] and we are interested in changes in the proportion of G2-M cells in the population, we adopted a generalized linear model (GLM) with binomial distribution and logit link using the R *glm* function to assess significance for the effect of SIP and bacterial exudates. *Post hoc* tests comparing all combinations of treatments were carried out using the *glht* function from the Multcomp package (Hothorn et al., 2008).

### RNA Extraction and Quality Assessment

The cells for RNA extraction (40 mL suspension, see above) were harvested by filtration over a Versapor filter (3 μm pore size, 25 mm diameter, PALL). Immediately after filtration, the filters were put in Eppendorf Tubes, flash-frozen in liquid nitrogen, and stored at −80°C until RNA extraction.

RNA was extracted from all samples (six treatments, five replicates each) using the RNeasy^®^ Plant Mini Kit (Qiagen). First, 1 mL RLT buffer and 10 μl β-mercaptoethanol were added to the Eppendorf tube containing the frozen filter. The cells were removed from the filter by pipetting up and down and using the pipet tip to scrape the filter. The filter was removed and silicon carbide beads (1 mm, BioSpec) were added to the Eppendorf Tube. The cells were lysed by silicon carbide beads beating on a beating mill (Retsch, 3 × 1 min at frequency 20 Hz, with 30 s on ice in between each run). The lysate was then transferred to a QIAshredder spin column (RNeasy^§^ kit) and the manufacturer’s instructions were followed from there. An on-column DNase treatment was performed using the RNase-free DNase set (Qiagen) according to the manufacturer’s instructions.

RNA quality was evaluated by spectrophotometry (Nanodrop^TM^) and Bioanalyzer (Agilent Technologies). For each treatment, the three replicates with the highest quality (high 260/230- and 280/260 ratio’s and high RIN-value) were selected for library preparation and sequencing.

### RNA Sequencing and Transcriptomic Analysis

The 18 sequencing libraries were prepared using Illumina^§^ TruSeq Stranded mRNA kit. The libraries were sequenced (2 × 75 bp) in one Illumina^®^ NextSeq 500 H150 run. Library preparation and sequencing were performed by VIB Nucleomics Core (VIB, Leuven).

Paired-end reads were quality-trimmed using FastQ Quality Filter from the FastX Toolkit v. 0.0.13^3^ using the following settings: −q 28, −p 30. Using the Salmon software tool in quasi-mapping mode (Patro et al., 2017), the quality-trimmed reads were mapped to an annotated genes model assembly of *S. robusta*. To generate the annotated assembly, Illumina paired-end reads and PacBio long reads were combined in a hybrid assembly approach and gene models were annotated using expression data as training for the BRAKER1 (Hoff et al., 2016) pipeline. Next, functional annotations for the *S. robusta* gene models were determined using three different strategies: (i) InterProScan v5.3 (Jones et al., 2014) was run to scan protein sequences for matches against the InterPro protein signature databases; (ii) eggNOG-mapper (Huerta-Cepas et al., 2017) was executed with DIAMOND mapping mode, based on eggNOG 4.5 orthology data (Huerta-Cepas et al., 2016); and (iii) AnnoMine (Vandepoele et al., 2013) was employed to retrieve consensus gene functional annotation from protein similarity searches [using DIAMOND v0.9.9.110 maximum (Buchfink et al., 2015), *e*-value 10*e*−05 against Swiss-Prot (Bairoch and Apweiler, 2000) database]. Gene ontology terms were retrieved from the results of the eggNOG-mapper.

The transcript-level abundances generated with Salmon were imported into R (v.3.4.4) and aggregated to gene level counts using the tximport package (Soneson et al., 2015). Genes with low overall counts [counts-per-million (CPM) < 1 in at least three samples] were removed from the libraries because they have little power for detecting differential expression (DE). Differences in sequencing depth and RNA population were corrected using a weighted trimmed mean of the log expression ratios (TMM) normalization (Robinson and Oshlack, 2010). Preliminary differences between expression profiles of different samples were explored with multi-dimensional scaling (MDS) plots based on the top 500 genes, generated using the plotMDS function included in the EdgeR package.

**FIGURE 1:**
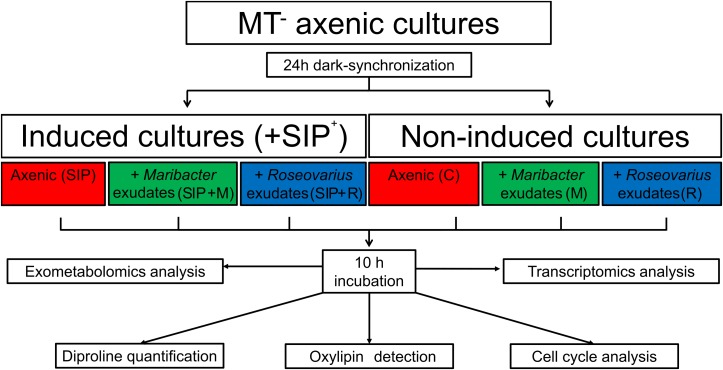
Experimental setup. Axenic MT^–^ *S. robusta* cells were grown in F/2 medium until an *F*_0_-value of ≈0.3. Their cell-cycle was dark-synchronized for 24 h in the darkness. After 21 h, half of the samples were treated with sexual inducing pheromone (SIP^+^) previously harvested from MT^+^. Bacterial exudates either from *Maribacter* sp. or *Roseovarius* sp. were also added. All samples were kept in the darkness for an additional 3 h before switching on the light. After 10 h of light, both cells and exudates from the diatom cultures were harvested. Cells were used for RNA extraction and cell cycle analysis, the medium was analyzed with an untargeted metabolomics approach and a targeted approach to detect diproline and oxylipins.

Differential expression analysis was performed using the R package edgeR 3.20.9 (Robinson et al., 2010). Negative binomial GLMs were fitted to model read counts for each gene in each sample and a dispersion parameter which accounts for variability between biological replicates was calculated (Lun et al., 2016). For DE analysis, nine comparisons (contrasts) were defined (SIP vs. C, M vs. C, R vs. C, SIP + M vs. SIP, SIP + R vs. SIP, SIP + R vs. R, SIP + M vs. M, SIP + M vs. SIP + R, see Figure 1 for experimental setup). A gene was considered differentially expressed (DE) if the false discovery rate (FDR) adjusted *p*-values were below 0.01 and the absolute log_2_ fold change (LFC) was equal or greater than 1. To confirm GTP specificity of the putative guanylate cyclases (GC), a multiple sequence alignment was carried out in MEGA 7 (Kumar et al., 2016) to check the presence of guanylate cyclase-specific motifs (Winger et al., 2008).

For genes DE in one specific contrast, Gene Ontology enrichment for single comparisons was determined using a gene set enrichment approach (GSEA) as implemented in CAMERA (Wu and Smyth, 2012), included in the R package limma v.3.34.9 (Ritchie et al., 2015). Redundant GO terms were removed using REVIGO^4^ (Supek et al., 2011) using a low similarity value of 0.5. GO enrichment of genes that were DE in multiple contrasts was performed using Fisher’s exact test and the “weight” algorithm for GO group scoring as implemented in TopGO (Alexa and Rahnenführer, 2009). Venn diagrams were generated with the R package VennDiagram v. 1.6.20 and with the web-based application Venny v. 2.1 (Oliveros, 2007–2015^5^).

### Exometabolome Extraction

A total of 150 mL of filtered medium from each culture flask was transferred to sterile and cleaned 250 mL Erlenmeyer flasks, which were covered immediately with aluminum foil and cooled down to 4°C before solid phase extraction. *Roseovarius* sp. and *Maribacter* sp. exudates (*n* = 4, diluted to an equivalent OD_600_ = 0.05 with minimal medium) were prepared and stored in the same way. Before extraction, 15 nmol of caffeine dissolved in methanol [HPLC grade, Sigma–Aldrich, Chromasolv^®^Plus (≥99.9%)] was added to each sample as an internal standard. The medium was extracted on 60 mg Oasis^®^ HLB-SPE cartridges (Waters, Eschborn, Germany), following the manufacturer’s instructions. Gentle vacuum was applied to the cartridges with a Visiprep^TM^ SPE Vacuum Manifold (Sigma–Aldrich) to have a flow-through of ca. 1 drop per second. The cartridges were eluted three times with 1 mL of methanol. The 3 mL of eluate was stored in 4 mL vial glass at −80°C until further analysis. Medium blanks (*n* = 3) were prepare in the same way by extracting sterile F/2 medium. 1.5 mL of the eluate from each sample was transferred to a clean vial, evaporated under a stream of nitrogen, and dissolved in 50 μl of methanol. Two quality control (QC) samples were prepared by pooling 5 μl from each sample in one clean vial.

### UHPLC-MS Measurements

After randomizing the measuring order list of the samples and including QC every 7 samples, 5 μl of each sample were analyzed by UHPLC Dionex UltiMate^®^ 3000 (Thermo Fisher Scientific, Dreieich, Germany), coupled to an ESI-Orbitrap MS Q-Exactive Plus (Thermo Fisher Scientific, Dreieich, Germany).

Liquid chromatography was performed on an Accucore^®^ C18 column (2.1 × 100mm, 2.6 μm particle size; Thermo Scientific, Dreieich, Germany). The composition of the mobile phase was set to 100% A (0.1% HCOOH and 2% ACN in H_2_O) for 0.2 min and ramped to 100% B (0.1% HCOOH in ACN) in a linear gradient within 9 min. The solvent composition was held at 100% B for 4 min, returned to 100% A in 0.1 min, and held at 100% A for 0.9 min. The flow rate ramped from 0.4 to 0.7 mL min^–1^ from 0.5 to 13.5 min.

Ionization was performed with a spray voltage of 3 kV and a capillary temperature of 360°C. Nitrogen was used as desolvation gas.

For monitoring, the scanned mass range was between 100 and 1,500 *m/z*, at a resolution *m*/Δ*m* 280,000 full-width at half maximum (FWHM) (*m/z* 200) in positive mode, with automatic gain control (ACG) target 3 × 10^6^, a maximum injection time (IT) of 200 ms.

For compound identification, full-scan MS/data-dependent MS/MS (ddMS^2^) experiment was performed on QC samples. Each experiment was composed of one full MS and up to 5 ddMS^2^. The five ions with the most intense signal detected in the full MS scan (intensity threshold 1.6 × 10^5^) produced a specific MS/MS spectrum. For full MS, the settings were the ones described above, while for the data-dependent MS/MS the settings were the following: positive mode with a resolution of *m*/Δ*m* 35,000 and an ACG target 1 × 10^5^, a maximum IT of 50 ms, a stepped normalized collision energy (NCE, 15, 30, 45), an isolation window of 0.4 *m/z*.

All data were acquired and processed with the software Xcalibur^TM^ version 3.0.63 (Thermo Fisher Scientific, Bremen, Germany).

### LC–HR–MS Data Analysis

Xcalibur^TM^ raw data files were imported into Thermo Compound Discoverer 2.1.0.398 (Thermo Fisher Scientific, Bremen, Germany) and analyzed following a standard pipeline for untargeted metabolomics for high resolution spectra. The important values for features extraction are the following: precursor ion deviation 5 ppm, maximum retention time shift 0.5 min, signal-to-noise threshold (S/N) 3, minimum peak intensity for peak selection 1 × 10^6^ au, retention time shift for grouping 0.5 min, and relative intensity tolerance for isotope search 30%. The exact masses of unknown compounds found in the samples were compared to online databases (PubChem, ChemSpider, mzCloud) and to an in-house library of 650 natural compounds (mass tolerance = 5 ppm) for identification.

After the analysis, a table with putative compound names and the molecular formula, exact masses, retention times, and chromatographic area for each sample was exported for further processing. All features found in the medium blank samples were removed from the samples. Data were then filtered based on QCs coefficient of variation (CV): only features with CV < 20% were retained (Dunn et al., 2011).

Finally, data were normalized on diatom biomass (using *F*_0_ as a proxy) and Pareto scaled to obtain normally distributed data. The obtained .csv table was used to perform statistical analysis with MetaboAnalystR (Chong and Xia, 2018). Principal component analysis (PCA) was performed to detect grouping and outliers in the samples. Significant features were selected from the results of one-way ANOVA analysis (FDR-adjusted *p*-value cutoff = 0.05, Fisher LSD *post hoc* analysis), which were visualized by heatmaps (distance measure = euclidean, clustering algorithm = Ward).

After statistical analysis, significant features were selected in the Thermo Compound Discoverer molecule list and exported to SIRIUS v. 4.0 (Böcker et al., 2009) to confirm features identity. Default settings for Orbitrap High Resolution Mass Spectrometry were used (ppm = 5), choosing all the possible adducts as candidates. For structural evaluation of compounds, CSI:FingerID (Dührkop et al., 2015) was used to compare to PubMed spectral database.

### Analysis of Oxylipins

Targeted detection of oxylipins was based on a method by Rettner et al. (2018). Briefly, measurements were performed on Acquity^TM^ UPLC system (Waters, Milford, MA, United States) coupled with a QTrap^®^ 5500 (ABSciex, Darmstadt, Germany). We used an ACQUITY UPLC^®^ BEH C18 column for separation (1.7 μm, 2.1 × 100 mm; Waters, Eschborn, Germany) kept at 50°C. The QTrap 5500 was operated in negative ionization mode only using scheduled multiple reaction monitoring (MRM). The scheduled MRM window was 60 s, and each oxylipin parameter was optimized individually (CE, EP, DP, CXP). The investigated oxylipins are the same analyzed by Rettner et al. (2018). Following instrument settings were used: curtain gas 35, collision gas medium (MRM); ion spray voltage −4,000, temperature 500°C, ion source gas 1 and 2 40. Solvent used were A: 100% H_2_O + 0.01% CH_3_COOH and B: 100% CH_3_OH + 0.01% CH_3_COOH with a solvent flow 0.3 mL ^*^ min^–1^. Injection volumes 10 μL. The gradient started at 42% B, ramped to 86% B at 12.5 min, then 98% B at 15.5 min, returned to 42% B in 0.5 min and re-equilibrated for 1 min. Manual integration of corresponding peaks was carried out using the Analyst software version 1.6. To confirm their presence in all samples, arachidonic acid and 15-hydroxyeicosatetraenoic acid (HETE) were additionally measured in negative mode on a UHPLC Dionex UltiMate^®^ 3000 (Thermo Fisher Scientific, Dreieich, Germany), coupled to an ESI-Orbitrap MS Q-Exactive Plus (Thermo Fisher Scientific, Dreieich, Germany), following the method mentioned in the previous paragraph. Identity of the compounds was confirmed by comparison with an external standard.

### Diproline Quantification Using GC–MS

Diproline was quantified following the method of Gillard et al. (2013). One microliter of the extract was injected into an ISQ Trace GC Ultra GC/MS system (Thermo Fisher, Dreieich, Germany) equipped with a 0.25 μm × 0.25 mm × 30 m DB-5MS + DG column (Agilent, Böblingen, Germany). Helium 5.0 (Linde AG, Pullach, Germany) was used as carrier gas with a constant flow of 1.2 mL min^–1^. The split ratio was 1:8. The initial oven temperature of 155°C was held for 3 min, ramped to 210°C with 25°C min^–1^, then to 255°C with 7°C min^–1^, and finally to 315°C with 25°C min^–1^, which was held for 3 min. The injector temperature was kept at 300°C during the entire run. The scanned mass range was between 50 and 400 *m/z* with a scan rate of 0.5 scans s^–1^ and an inter-scan delay of 0.04 s^–1^. Electron ionization was carried out at 70 eV.

To quantify diproline, the peak area of both diproline and internal standard (caffein, 15 nmol) was determined with the function Quan Browser included in the software Xcalibur^TM^ version 3.0.63 (Thermo Fisher Scientific, Bremen, Germany) and Microsoft Office Excel (Microsoft^®^, United States). All results were normalized to the diatom biomass. A one-way ANOVA followed by Bonferroni’s multiple comparisons tests was performed using GraphPad Prism version 7.00 for Windows (GraphPad Software, La Jolla, CA, United States^6^).

## Results and Discussion

### Bacterial Exudates Do Not Influence the Diatom Cell Cycle Arrest During Sexual Reproduction

In order to maximize sexual reproduction success, diatoms need to finely synchronize their cell cycle. When a suitable mating partner is present, *S. robusta* cell cycle is temporarily arrested in G1 phase by the SIP of the opposite mating type, resulting in a synchronized switch from a mitotic to meiotic cell cycle in both partners (Gillard et al., 2013; Moeys et al., 2016). Some studies reported that algicidal bacteria can have an effect on microalgal cell cycle progression (Pokrzywinski et al., 2017). We therefore tested if the effect of bacterial exudates on sexual reproduction observed by Cirri et al. (2018) is due to an interference with the regulation of the cell cycle during the initial sexual stages.

The relative number of MT^–^ cells in G1- and S/G2-phase of the cell cycle was measured in six different treatments after dark-synchronization in G1 phase (Figure 1): control (non-induced, axenic, C), induced cultures (axenic, SIP), *Roseovarius* sp. exudates + non-induced cultures (R), *Maribacter* sp. exudates + non-induced cultures (M), *Roseovarius* sp. exudates + induced cultures (SIP + R), and *Maribacter* sp. exudates + induced cultures (SIP + M).

**FIGURE 2:**
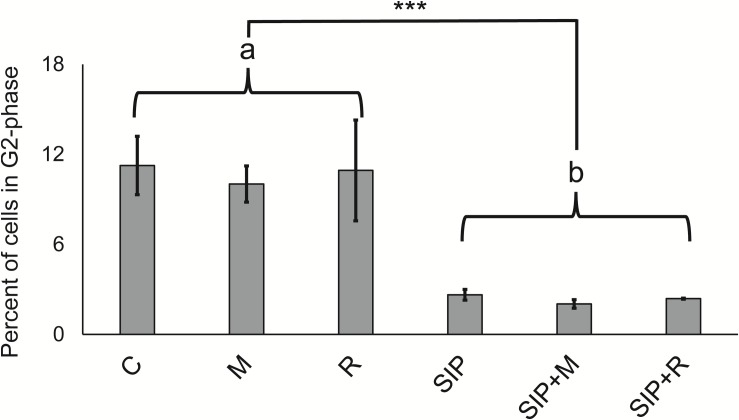
Cell cycle analysis. Flow cytometric measurements of the percentages of cells that have progressed through S-phase for all six experimental treatments. C is the axenic, non-induced control; M is the non-induced control + *Maribacter* sp. exudates; R is the non-induced control + *Roseovarius* sp. exudates; SIP is the induced axenic control; SIP + M is the induced culture + *Maribacter* sp. Exudates; SIP + R is the induced control + *Roseovarius* sp. exudates. Proportion post-S-phase cells differed significantly between all non-conditioned cells (“a”) and SIP^+^-conditioned cells (“b”). ^*^p < 0.05, ^∗∗^*p* < 0.01, and ^∗∗∗^*p* <0.001.

Ten hours after re-illumination, the percentage of cells in S/G2 phase was significantly lower (*p* < 0.001) in the SIP-induced cultures compared to non-induced controls, confirming that SIP^+^ arrests cell cycle progression of MT^–^ in G1 phase (Moeys et al., 2016; Figure 2). The presence of exudates only (without SIP^+^ induction) did not reduce the peak in S/G2-phase cells, suggesting that the bacterial exudates did not affect the cell cycle progression in mitotic cells. More importantly, *post hoc* contrasts (SIP vs. SIP + M and SIP vs. SIP + R) did not show significant effects of exudates on cell cycle progression compared with SIP^+^-treated cultures (*p* = 0.8 and *p* = 0.91, respectively). Therefore, we conclude that the effect of bacterial exudates on sexual reproduction in *S. robusta* is not due to interference with the SIP^+^-induced cell cycle arrest.

### Bacterial Exudates Do Not Influence Sexual Reproduction Processes of *S. robusta*

To study the transcriptional changes in *S. robusta* MT^–^ cells in response to the presence of bacterial exudates, we extracted mRNA of induced and non-induced diatom cultures; both untreated and treated with bacterial exudates after 24 h dark-synchronization followed by 10 h of illumination (Figure 1). We obtained expression data for 25,557 genes. 4,225 unique genes (16.6% of the expressed genes) were DE in at least one treatment (Table 1, |LFC| > 1, FDR < 0.05) and more than half of these genes were functionally annotated (>59% in each comparison).

**TABLE 1:** Summary of the number of significantly differentially expressed genes in different comparisons.

	**SIP vs. C**	**SIP + M vs. M**	**SIP + R vs. R**	**M vs. C**	**SIP + M vs. SIP**	**R vs. C**	**SIP + R vs. SIP**
Up	983	484	613	268	406	105	180
Not sign.	22,305	23,716	23,344	25,226	25,027	25,450	25,367
Down	2,269	1,357	1,600	63	124	2	10

**FIGURE 3:**
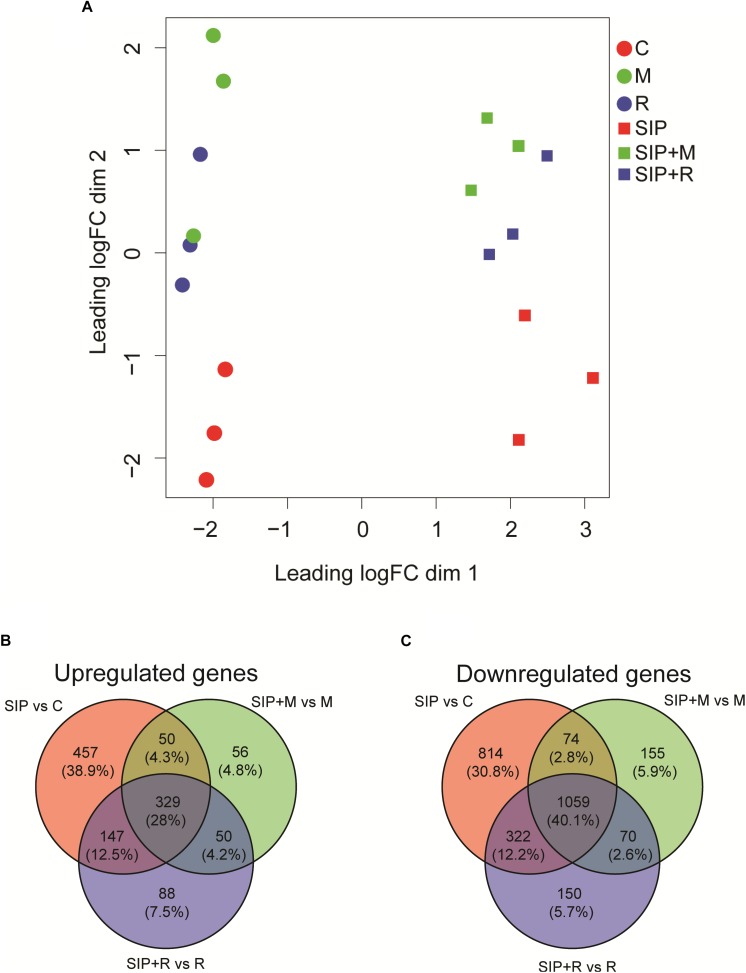
**(A)** Multi-dimensional scaling (MDS) plot for the obtained transcriptomes. Distance between samples is based on log_2_ fold changes. C is the axenic non-induced control; M is the non-induced control + *Maribacter* sp. exudates; R is the non-induced control + *Roseovarius* sp. exudates; SIP is the induced axenic control; SIP + M is the induced culture + *Maribacter* sp. exudates; SIP + R is the induced control + *Roseovarius* sp. exudates. **(B,C)** Venn diagrams of SIP^+^-induced up- **(B)** and downregulated **(C)** *S. robusta* genes. The up- and downregulated genes thresholds are: log_2_ fold change (LFC) = 1, false discovery rate (FDR) = 0.05.

A MDS plot of the differences in gene expression profiles between RNA-seq samples (Figure 3A) showed that the strongest difference in gene expression between samples was caused by the induction of sexuality (SIP^+^-treatment). This was confirmed by the high number of DE genes in induced cultures compared to non-induced cultures (SIP vs. C, SIP + M vs. M, and SIP + R vs. R: Table 1 and Supplementary Tables S1, S2). Moreover, in the comparisons of non-induced control cultures (C), non-induced cultures treated with *Maribacter* sp. exudates (M), and non-induced cultures treated with *Roseovarius* sp. exudates (R) with their SIP^+^-treated equivalents (SIP, SIP + M and SIP + R, respectively), high amounts of genes that were up- or downregulated in response to SIP^+^ were shared in all three comparisons (28% of the total upregulated genes and 40.1% of the total downregulated genes are shared in all three comparisons, Figures 3B,C).

Of this shared set of 329 genes that are SIP^+^-upregulated despite bacterial exudates presence (Figure 3B), some are associated to early meiosis-related processes (Table 2), especially dsDNA break repair, DNA duplex unwinding, and DNA replication (Supplementary Table S1: GO enrichment results). In conclusion, we show that of the known SIP^+^-triggered processes, early meiosis is not significantly affected by either bacterium.

**TABLE 2:** Upregulated genes involved in sexual reproduction and diproline production shared by all SIP+-induced cultures compared to non-induced controls (SIP vs. C, SIP + M vs. M, and SIP + R vs. R).

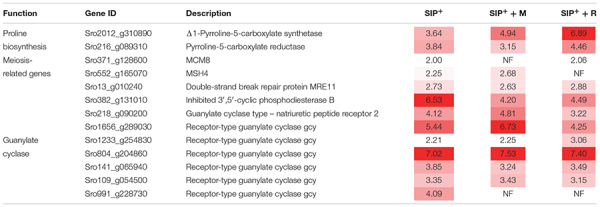

*Meiotic-related genes explanation: *MRE11* (Sro13_g010240) is part of the Mre11–Rad50–Nbs1 complex involved in repairing DNA double-strand breaks and homologous recombination during meiosis (Ajimura et al., 1993). *MSH4* (Sro552_g165070) is a meiosis-specific mismatch repair protein (Kolas and Cohen, 2004). *MCM8* (Sro371_g128600) is involved in meiotic recombination (Blanton et al., 2005). Red gradient indicates the different upregulation magnitude, indicated by the fold change number.*

### Receptor-Type Guanylate Cyclases May Be Involved in Diatom–Bacteria Recognition

We also found upregulation of genes involved in cGMP biosynthesis (GC) and breakdown (phosphodiesterases, PDE) (Table 2 and Supplementary Table S1). It has been shown that cGMP signaling likely plays an important role as a secondary messenger during the onset of sexual reproduction in pennate diatoms (Moeys et al., 2016; Basu et al., 2017). The upregulation of these genes was not uniform across the experimental treatments (Table 2), with some GC and PDE genes showing higher upregulation in axenic conditions (Sro991_g228730, LFC = 4.09) while others being more upregulated either in presence of *Roseovarius* sp. exudates (Sro1233_g254830) or in presence of *Maribacter* sp. exudates (Sro218_g090200, Sro1656_g289030). Interestingly, expression of several receptor-type GCs with PDE activities (GC/PDEs) was triggered by *Maribacter* sp. exudates (upregulation of seven GCs SIP + M vs. SIP, two of which contain a PDE domain, Supplementary Table S7). These receptor-type GCs were not DE in axenic conditions or in presence of *Roseovarius* sp. exudates, suggesting a role for specific cGMP-related signaling pathways during the perception of *Maribacter* sp. It has been shown that cyclic nucleotide signaling is crucial for an array of physiological processes in diatoms, from regulation of silicon cycle (Aline et al., 1984; Smith et al., 2016) to acclimation to CO_2_ (Hennon et al., 2015). Moreover, this mechanism was also suggested to be involved during the onset of the sexual reproduction in the diatom *Pseudo-nitzschia multistriata* (Basu et al., 2017). In plants, signaling by cyclic nucleotides (cGMP and cAMP) is well studied (Isner and Maathuis, 2018) and cAMPs were suggested to play a role in plant–bacteria interactions (Tian et al., 2012). In diatoms or other algae, a similar role of cGMP in inter-kingdom crosstalk has not been described so far. Our results suggest that these pathways may be involved in either the diatom/bacteria recognition process, or in the negative modulation of reproduction by *Maribacter* sp.

### *Maribacter* sp. Exudate Causes Major Changes in the *S. robusta* Gene Expression

The second major separation in gene expression profiles of *S. robusta* observed in the MDS plot corresponds to the presence or absence of bacterial exudates in MT^–^ cultures (Figure 3A). The replicates of induced samples treated with bacterial exudates (SIP + M and SIP + R) clustered together more closely compared to the replicates of non-induced samples (M and R), suggesting that the transcriptional changes caused by the bacterial exudates were more coherent when SIP^+^ is present. Additionally, the number of DE genes in response to the bacterial exudates was higher in the presence of SIP^+^ (Table 1: compare M vs. C, 331 DE genes with SIP + M vs. SIP, 530 DE genes; and compare R vs. C, 107 DE genes with SIP + R vs. SIP, 190 DE genes). Moreover, there is only limited overlap between genes that are DE in response to bacterial exudates in presence and absence of SIP^+^ (Supplementary Figure S2).

**FIGURE 4:**
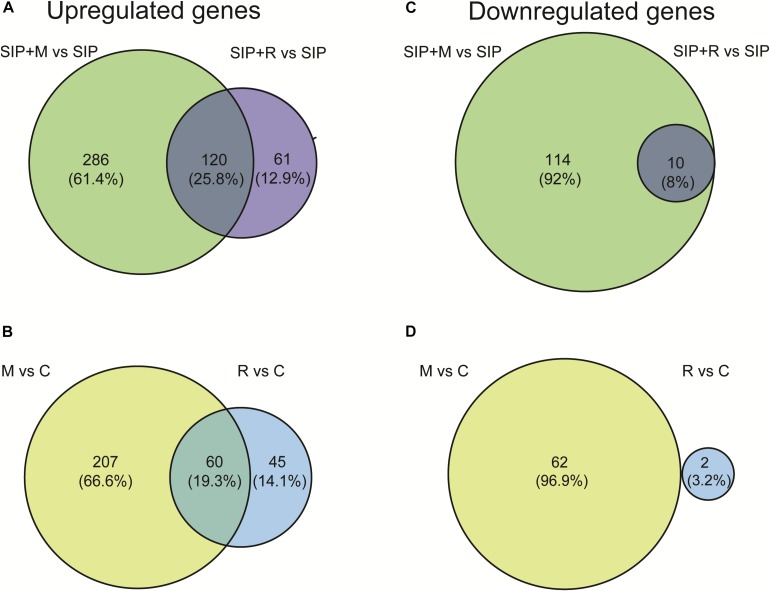
Venn diagrams showing overlap between *Maribacter* sp. (M)- and R*oseovarius* sp. (R)-induced DE genes in presence **(A,B)** and absence **(B,C)** of SIP^+^. **(A,B)** Upregulated **(A)** and downregulated **(B)** genes in response to *Maribacter* sp. (SIP + M) and *Roseovarius* sp. (SIP + R) treatments in presence of SIP^+^. **(C,D)** Upregulated **(C)** and downregulated **(D)** genes in response to *Maribacter* sp. (M) and *Roseovarius* sp. (R) treatments in absence of SIP^+^. The up- and downregulated genes thresholds are: log_2_ fold change (LFC) = 1, false discovery rate (FDR) = 0.05.

Because *Maribacter* sp. and *Roseovarius* sp. affect sexual reproduction of *S. robusta*, albeit in opposite directions (Cirri et al., 2018), we next focused on transcriptional changes observed in induced S. *robusta* in the presence and absence of bacterial exudates (SIP + M vs. SIP and SIP + R vs. SIP). Venn diagrams showing the numbers of shared and unique up- and downregulated genes between SIP + M vs. SIP and SIP + R vs. SIP are, respectively, shown in Figures 4A,B, while Venn diagrams in Figures 4C,D display up- and downregulated genes in M vs. C and R vs. C, respectively. A detail description of up- and downregulated genes in the different treatments of induced *S. robusta* cultures is reported in Supplementary Tables S3, S5, S7, S8, S10.

Both in induced and in non-induced cultures, *Maribacter* sp. exudates triggered significantly more DE genes compared to *Roseovarius* sp. exudates (Table 1, Figure 4, and Supplementary Figures S1, S2). This indicates that *Maribacter* sp. has a stronger effect on the physiology of *S. robusta* compared to the *Roseovarius* sp.

### Proline Biosynthesis Genes Are Upregulated in Presence of *Roseovarius* sp. Exudates and Diproline Concentration Decreases in Presence of *Maribacter* sp. Exudates

One of our main research questions is whether the positive effect of *Roseovarius* and negative effect of *Maribacter* on sexual reproduction is linked to a change in diproline biosynthesis by the diatom. Moeys et al. (2016) hypothesized that the upregulation of proline biosynthesis is crucial for diproline synthesis, thereby increasing the proline pool that can be used for diproline production.

**FIGURE 5:**
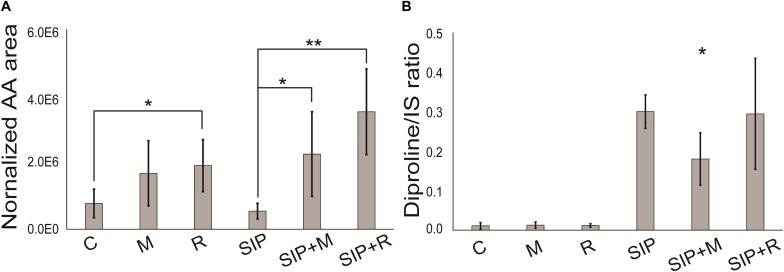
**(A)** Diproline and **(B)** arachidonic acid relative concentration. Arachidonic acid is normalized to diatom biomass. Diproline is normalized to diatom biomass and internal standard. Internal standard (IS) is caffeine (15 nmol). Significance was tested with a one-way ANOVA (adjusted *p*-value after Bonferroni’s correction for multiple comparisons = 0.05). SIP^+^ axenic treatment is taken as control for assessing significant differences in diproline concentration in SIP + M and SIP + R treatments. C is the axenic, non-induced control; M is the non-induced control + *Maribacter* sp. exudates; R is the non-induced control + *Roseovarius* sp. exudates; SIP is the induced axenic control; SIP + M is the induced culture + *Maribacter* sp. exudates; SIP + R is the induced control + *Roseovarius* sp. exudates. ^*^p < 0.05, ^∗∗^*p* < 0.01, and ^∗∗∗^*p* <0.001.

Δ1-Pyrroline-5-carboxylate synthetase (*P5CS*, Sro2012_g310890), a key enzyme in proline biosynthesis (Hu et al., 1992), was upregulated in SIP vs. C, SIP + M vs. M, and SIP + R vs. R, but the strongest upregulation was observed in presence of *Roseovarius* sp. exudates (LFC = 6.89, FDR < 10^–6^), while upregulation was less strong in axenic conditions (LFC = 3.64, FDR < 10^–4^) or in presence of *Maribacter* sp. exudates (LFC = 4.94, FDR < 10^–5^) (Table 2). Another gene of this pathway, Δ1-pyrroline-5-carboxylate reductase (*PC5*, Sro216_g089310), was also upregulated in all three comparisons. Here too, upregulation was stronger in the presence of *Roseovarius* sp. exudates (LFC = 4.46, FDR < 10^–5^) compared to *Maribacter* sp. exudates (LFC = 3.15, FDR < 10^–3^) or axenic conditions (LFC = 3.84, FDR < 10^–4^) (Table 2). To check if this gene regulation affects diproline production, we measured the attraction pheromone concentration in the medium of induced cultures both in presence and absence of the *Maribacter* sp. and *Roseovarius* sp. exudates (Figure 5A). These measurements confirmed that diproline is only produced in induced cultures. In absence of SIP^+^, the bacterial exudates did not trigger diproline production. Furthermore, as suggested by the transcriptome data, an increase in diproline concentration after treatment with *Roseovarius* sp. exudates occurs; however, the increase is small and not significant (Figure 5A). More interestingly, we observed that the diproline production in induced cultures was significantly lower in the presence of *Maribacter* sp. exudates (*p* < 0.012) compared to axenic cultures. Our transcriptomic data reveal that the proline biosynthetic pathway was strongly upregulated in all induced cultures. Since conditioning with SIP^+^ induces diproline production, this upregulation in response to all treatments with SIP^+^ supports the hypothesis by Moeys et al. (2016) that an increased proline production feeds into the diproline production pathway. Interestingly, diproline biosynthetic genes are upregulated most strongly in the presence of *Roseovariu*s sp. exudates, which is in accordance with the slightly higher concentration of diproline and the enhanced reproduction success observed by Cirri et al. (2018). On the other hand, induced cultures in presence of *Maribacter* sp. exudates did not show a decrease in gene expression of these genes. Thus, the lower concentration of diproline and the negative effect of *Maribacter* sp. on sexual reproduction cannot be explained through this pathway.

Considering that the exact mechanism of the attraction pheromone biosynthesis remains unknown and that a significant amount of *S. robusta* genes lack a good annotation, it is possible that other but yet unknown pathways related to diproline production are involved.

### Gene Expression Changes of Amino Acid and Photosynthesis-Related Enzymes in Response to *Maribacter* sp. Exudates Are Potentially Altering the Pool of Proline Precursors in the Cell

Gene ontology enrichment of a set of genes that is upregulated in induced culture only when *Maribacter* sp. exudates are present (SIP + M vs. SIP, Supplementary Table S7) showed a downregulation of several amino acid catabolic pathways, especially those of tyrosine (two genes), arginine (two genes), and phenylalanine (two genes) (Supplementary Table S9). The downregulation of these pathways was stronger in presence of SIP^+^ (SIP + M vs. SIP, Table 4): four downregulated genes involved in tyrosine metabolism, four for phenylalanine catabolism, and two for arginine catabolism. Downregulation in response to *Maribacter* sp. exudates was strongest for a tyrosine aminotransferase (Sro379_g130480) and two fumarylacetoacetase (Sro341_g121520 and Sro341_g121510) (LFC < −3.9, LFC < −3.4, and LFC < −3.33, respectively, in SIP + M vs. SIP, Supplementary Table S8). Both are involved in phenylalanine catabolism: the former enzyme catalyzes the conversion of tyrosine to 4-hydroxyphenylpyruvate, the latter breaks down fumarylacetoacetate into fumarate and acetoacetate (Santucci et al., 2017), thus influencing the TCA cycle. Interestingly, the phenylalanine-to-tyrosine pathway was one of the processes that was actively upregulated by SIP^+^ (Supplementary Table S1: phenylalanine 4-monooxygenase activity). In higher plants, phenylalanine and tyrosine are produced via the shikimate pathway (Tzin and Galili, 2010) and it has been suggested that downstream products like tyramine are involved in defense responses (Trezzini et al., 1993). In diatoms, less is known about the importance of the metabolism of these two amino acids. However, their biosynthesis is strongly connected to the biosynthetic pathway of tryptophan (Bromke, 2013), an amino acid that has a fundamental role in algae–bacteria interactions (Amin et al., 2015).

Interestingly, in cultures treated with SIP^+^ and *Maribacter* sp. exudates, a total of 40 genes associated with photosynthetic functions and the light-harvesting complex (LHC) were upregulated compared to the SIP^+^ only treatment (SIP + M vs. SIP), many of which were downregulated in SIP vs. Control (Table 3 and Supplementary Table S7). Twenty-two of these were fucoxanthin-chlorophyll a–c binding proteins (FCPs, Supplementary Table S7), intrinsic proteins of the thylakoid membrane that bind chlorophyll a and c and that are responsible for the absorption of the blue–green wavelengths in aquatic environments (Schellenberger Costa et al., 2012; Kuczynska et al., 2015). FCPs are also involved in non-photochemical quenching (NPQ) (Kuczynska et al., 2015), a mechanism that protects plants and algae from high light stress (Horton and Ruban, 2004; Dong et al., 2016). So far, nothing was known about possible effects of bacteria on diatom FCPs or NPQ, and the biological significance of this observation requires more in-depth photophysiological studies. Next to the FCP genes, we identified four genes involved in carotenoid and chlorophyll biosynthesis which are upregulated in SIP + M vs. SIP: a carotene desaturase (Sro536_g162170), a glutamate tRNA ligase (Sro20_g014070), and two glutamate-1-semialdehyde 2,1-aminomutases (Sro479_g151140 and Sro1597_g284880) (Supplementary Table S7). The strong upregulation of these enzymes, combined with the downregulation of the arginine catabolic pathway (Table 4), could diminish the availability of glutamate and arginine, two important substrates for proline biosynthesis in diatoms (Bromke, 2013).

**TABLE 3:** GO enrichment of genes upregulated by *Maribacter* sp. in the presence of SIP^+^ (SIP + M vs. SIP).

**Upregulated in response to *Maribacter* sp. in presence of SIP^+^**			
**GO ID**	**Description**	**Genes**	***p*-value**

**Biological functions**			
GO:0006779	Porphyrin-containing compound biosynthetic process	9	8.40*E*−09
GO:0009768	Photosynthesis, light harvesting in photosystem I	4	2.50*E*−05
GO:0015994	Chlorophyll metabolic process	5	2.70*E*−05
GO:0010218	Response to far red light	4	4.60*E*−04
GO:0010114	Response to red light	4	8.40*E*−04
GO:0016116	Carotenoid metabolic process	3	1.44*E*−03
GO:0055114	Oxidation–reduction process	23	1.79*E*−03
GO:0009637	Response to blue light	4	2.43*E*−03
GO:0042374	Phylloquinone metabolic process	2	3.43*E*−03
GO:0070127	tRNA aminoacylation for mitochondrial protein translation	2	6.27*E*−03
GO:0000103	Sulfate assimilation	2	7.98*E*−03
GO:0031388	Organic acid phosphorylation	1	1.55*E*−02
GO:0019424	Sulfide oxidation, using siroheme sulfite reductase	1	1.55*E*−02
GO:0007225	Patched ligand maturation	1	1.55*E*−02
GO:0042049	Cellular acyl-CoA homeostasis	1	1.55*E*−02
GO:0009704	De-etiolation	1	1.55*E*−02
GO:0006427	Histidyl-tRNA aminoacylation	1	1.55*E*−02
GO:1900160	Plastid DNA packaging	1	1.55*E*−02
**Molecular functions**			
GO:0004783	Sulfite reductase (NADPH) activity	2	0.00023
GO:0042286	Glutamate-1-semialdehyde 2,1-aminomutase activity	2	2.30*E*−04
GO:0016634	Oxidoreductase activity, acting on the CH–CH group of donors, oxygen as acceptor	2	2.22*E*−03
GO:0010181	FMN binding	2	3.30*E*−03
GO:0004500	Dopamine beta-monooxygenase activity	2	1.15*E*−02
GO:0050311	Sulfite reductase (ferredoxin) activity	1	1.52*E*−02
GO:0004853	Uroporphyrinogen decarboxylase activity	1	1.52*E*−02
GO:0015390	Purine-specific nucleoside:sodium symporter activity	1	1.52*E*−02
GO:0050561	Glutamate-tRNA(Gln) ligase activity	1	1.52*E*−02
GO:0004631	Phosphomevalonate kinase activity	1	1.52*E*−02
GO:0004821	Histidine-tRNA ligase activity	1	1.52*E*−02
GO:0030248	Cellulose binding	1	1.52*E*−02
GO:0004160	Dihydroxy-acid dehydratase activity	1	1.52*E*−02
GO:0015389	Pyrimidine- and adenine-specific:sodium symporter activity	1	1.52*E*−02
GO:0016162	Cellulose 1,4-beta-cellobiosidase activity	1	1.52*E*−02
GO:0047012	Sterol-4-alpha-carboxylate 3-dehydrogenase (decarboxylating) activity	1	1.52*E*−02
GO:0008685	2-C-methyl-D-erythritol 2,4-cyclodiphosphate synthase activity	1	1.52*E*−02
GO:0016002	Sulfite reductase activity	1	1.52*E*−02
GO:0009976	Tocopherol cyclase activity	1	1.52*E*−02
GO:0003864	3-Methyl-2-oxobutanoate hydroxymethyltransferase activity	1	1.52*E*−02
GO:0003854	3-Beta-hydroxy-delta5-steroid dehydrogenase activity	1	1.52*E*−02
GO:0000252	C-3 sterol dehydrogenase (C-4 sterol decarboxylase) activity	1	1.52*E*−02
GO:0050421	Nitrite reductase (NO-forming) activity	1	1.52*E*−02
**Cellular component**			
GO:0044434	Chloroplast part	55	6.60*E*−29
GO:0009337	Sulfite reductase complex (NADPH)	2	2.70*E*−04
GO:0048046	Apoplast	7	2.80*E*−04
GO:0020011	Apicoplast	8	9.30*E*−04
GO:0009509	Chromoplast	2	3.89*E*−03

**TABLE 4:** GO enrichment of genes downregulated by *Maribacter* sp. in the presence of SIP^+^ (SIP + M vs. SIP).

**Downregulated in response to *Maribacter* sp. in presence of SIP^+^**			
**GO ID**	**Description**	**Genes**	***p*-Value**

**Biological function**			
GO:0009083	Branched-chain amino acid catabolic process	7	5.20*E*−12
GO:0006559	L-Phenylalanine catabolic process	4	2.20*E*−08
GO:0006570	Tyrosine metabolic process	4	1.00*E*−07
GO:0051262	Protein tetramerization	4	3.20*E*−05
GO:1902000	Homogentisate catabolic process	2	5.70*E*−05
GO:0006637	Acyl-CoA metabolic process	4	1.10*E*−04
GO:0006527	Arginine catabolic process	2	2.80*E*−04
GO:0006567	Threonine catabolic process	2	4.00*E*−04
GO:0033539	Fatty acid beta-oxidation using acyl-CoA dehydrogenase	2	5.30*E*−04
GO:0000098	Sulfur amino acid catabolic process	2	2.50*E*−03
GO:0010188	Response to microbial phytotoxin	1	4.45*E*−03
GO:0044524	Protein sulfhydration	1	4.45*E*−03
GO:0018272	Protein-pyridoxal-5-phosphate linkage via peptidyl-N6-pyridoxal phosphate-L-lysine	1	4.45*E*−03
GO:0008205	Ecdysone metabolic process	1	4.45*E*−03
GO:0007563	Regulation of eclosion	1	4.45*E*−03
GO:0009684	Indoleacetic acid biosynthetic process	1	8.89*E*−03
GO:0002047	Phenazine biosynthetic process	1	8.89*E*−03
GO:0019343	Cysteine biosynthetic process via cystathionine	1	8.89*E*−03
GO:0046951	**Ketone body biosynthetic process**	1	8.89*E*−03
GO:0001560	Regulation of cell growth by extracellular stimulus	1	8.89*E*−03
GO:0019346	Transsulfuration	1	1.33*E*−02
**Molecular functions**			
GO:0004485	Methylcrotonyl-CoA carboxylase activity	2	2.10*E*−05
GO:0016937	Short-branched-chain-acyl-CoA dehydrogenase activity	2	2.10*E*−05
GO:0004085	Butyryl-CoA dehydrogenase activity	2	6.20*E*−05
GO:0004334	Fumarylacetoacetase activity	2	6.20*E*−05
GO:0004121	Cystathionine beta-lyase activity	2	6.20*E*−05
GO:0016833	Oxo-acid-lyase activity	2	1.20*E*−04
GO:0050897	Cobalt ion binding	2	4.51*E*−03
GO:0044540	L-Cystine L-cysteine-lyase (deaminating)	1	4.64*E*−03
GO:0047982	Homocysteine desulfhydrase activity	1	4.64*E*−03
GO:0080108	*S*-alkylthiohydroximate lyase activity	1	4.64*E*−03
GO:0033855	Nicotianamine aminotransferase activity	1	4.64*E*−03
GO:0047022	7-Beta-hydroxysteroid dehydrogenase (NADPH) activity	1	4.64*E*−03
GO:0004505	Phenylalanine 4-monooxygenase activity	1	4.64*E*−03
GO:0004490	Methylglutaconyl-CoA hydratase activity	1	4.64*E*−03
GO:0034617	Tetrahydrobiopterin binding	1	4.64*E*−03
GO:0004838	L-Tyrosine:2-oxoglutarate aminotransferase activity	1	4.64*E*−03
GO:0008709	Cholate 7-alpha-dehydrogenase activity	1	4.64*E*−03
GO:0001540	Amyloid-beta binding	1	9.26*E*−03
GO:0004474	Malate synthase activity	1	9.26*E*−03
GO:0004658	Propionyl-CoA carboxylase activity	1	9.26*E*−03
GO:0004303	Estradiol 17-beta-dehydrogenase activity	1	9.26*E*−03
GO:0009374	Biotin binding	1	1.39*E*−02
GO:0008418	Protein-*N*-terminal asparagine amidohydrolase activity	1	1.39*E*−02
GO:0004123	Cystathionine gamma-lyase activity	1	1.39*E*−02
GO:0033938	1,6-Alpha-L-fucosidase activity	1	1.39*E*−02
**Cellular component**			
GO:0005759	Mitochondrial matrix	8	9.00*E*−06
GO:0012511	Monolayer-surrounded lipid storage body	1	1.30*E*−02

**FIGURE 6:**
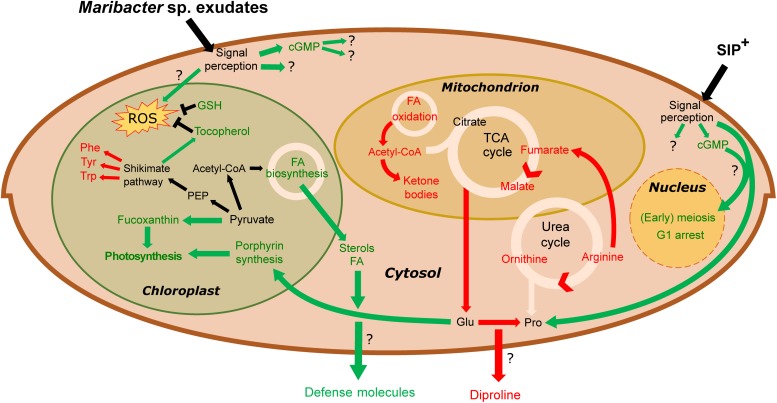
Overview of metabolic changes in *S. robusta* when exposed to SIP^+^ and *Maribacter* sp. exudates. In green are the upregulated processes, red the downregulated ones. *Maribacter* sp. exudates do not directly influence early meiotic processes. Stress induced by bacterial exudates triggers cGMP signaling cascades, an upregulation of photosynthetic pigment production and an oxidative stress response (by tocopherol and glutathione biosynthesis). Flux through the urea- and TCA cycle is reduced, diminishing intracellular arginine, fumarate, malate, and glutamate pools. Glutamate, precursor for proline synthesis, is used for porphyrin production, so the upregulated porphyrin synthesis could affect proline biosynthesis and thus also diproline production. Phe, phenylalanine; Tyr, tyrosine; Trp, tryptophan; Glu, glutamate; Pro, proline; GSH, glutathione; FA, fatty acid; PEP, phosphoenolpyruvate.

Taking these results into account, it appears that treatment with *Maribacter sp.* exudates has a strong influence on gene expression of amino acid metabolism and LHC genes. We observed that *Maribacter* sp. exudates do not negatively influence the sexual reproduction of *S. robusta* by directly targeting proline production. Instead, we hypothesize that the upregulation of photosynthetic pigment production, combined with the diminishing glutamate availability might reduce the intracellular pool of proline precursors (glutamate, arginine) and thereby indirectly influences diproline biosynthesis (Figure 6). Contrary, in *Roseovarius* sp.-treated samples, we do observe an upregulation in proline biosynthetic genes and no upregulation of LHC-related genes (see Supplementary Tables S3–S6). This could result in an increased or prolonged diproline production and release, explaining the enhancement of sexual efficiency observed by Cirri et al. (2018) and the concentration of diproline comparable to that of axenic cultures.

### Both Bacterial Exudates Trigger Detoxification, Oxidative Stress Responses, and Oxylipins Precursor Release in *S. robusta*

Apart from transcriptional changes in *S. robusta* that were specific to the exudates produced either by *Maribacter* sp. or *Roseovarius* sp., both bacterial exudates caused upregulation of metabolic processes related to oxidative stress responses, detoxification, and defense mechanisms (Supplementary Tables S10, S11).

Several genes that were upregulated in response to both *Roseovarius* sp. and *Maribacter* sp. exudates in the presence of SIP^+^ encode proteins that contain a flavodoxin-like fold, as a NADPH-dependent oxidoreductase (Sro481_g151580, LFC > 7) and an alcohol dehydrogenase (Sro989_g228490, LFC > 5) (Supplementary Table S10). These proteins are involved in energy metabolism, electron transfer, and in response mechanisms to reactive oxygen species (ROS)-stimulated stress (Quijano et al., 2016; Sies et al., 2017; Poirier et al., 2018).

Moreover, both bacterial exudates influenced glutathione metabolism. Glutathione is a tripeptide acting as fundamental antioxidant in many eukaryotes, including phytoplankton (Poirier et al., 2018). Glutathione *S*-transferases (GST) (Sro1751_g295250 and Sro945_g223090) and glutathionyl-hydroquinone reductases (GS-HQR) (Sro596_g172810 and Sro2126_g315740) were found to be especially upregulated (Supplementary Table S10). These enzymes play important roles in detoxification reactions in plants. GSTs transfer GSH to electrophilic centers of toxic, hydrophobic compounds, and the resulting conjugates are more soluble and therefore less toxic (Sheehan et al., 2001). GS-HQRs are a particular type of GSTs that reduce GS-hydroquinones and are believed to play a maintenance role for an array of metabolic pathways in photosynthetic organisms (Belchik and Xun, 2011).

Furthermore, sterol and fatty acid biosynthetic pathways were affected by the presence of both bacterial exudates. Cholesterol catabolism and the concomitant upregulation of tocopherol cyclase activity (Supplementary Table S11) indicated that *S. robusta* may use this molecule as a defense mechanism against oxidative stress. Tocopherols are antioxidants present in plastids of all lineages of photosynthetic eukaryotes and are involved in different stress responses in diatoms (Havaux and García-Plazaola, 2014; Lauritano et al., 2015). Fatty acid catabolism (fatty acid beta-oxidation) and ketone body synthesis were particularly influenced by *Maribacter* sp. exudates. Acetyl-CoA metabolism (Table 4) and fumarylacetoacetase activity (Table 4) were downregulated, leading to a decreased fumarate pool, involved in the TCA cycle. Also enoyl-CoA hydratase (Sro2125_g315680, LFC < −3.3, Supplementary Table S8), an enzyme responsible for hydrating the double bond between the second and third carbons of Acyl-CoA and involved in fatty acid catabolism to produce acetyl-CoA and energy (Bahnson et al., 2002), was downregulated. All these observations suggest that, in the presence of bacteria exudates, *S. robusta* metabolism shift from fatty acids catabolism to intracellular accumulation of this compounds (Shi and Tu, 2015), maybe to function as defense mechanisms. The detection of upregulated acyl-CoA metabolic pathways in presence of *Maribacter* sp. exudates (SIP + M vs. SIP, Supplementary Table S11), different from the downregulated acyl-CoA pathways mentioned above, supports this hypothesis. Interestingly, a putative 12-oxophytodienoate reductase (OPR) (Sro250_g098890) was strongly upregulated in induced cultures when both bacterial exudates were present (LFC > 6) (Supplementary Table S10). OPRs are flavoprotein enzymes that regulate jasmonic acid biosynthesis from the fatty acid linolenic acid, a crucial mediator of chemical defense mechanisms and plant–microbe interactions in plants (Erb, 2018; Koo, 2018). More generally, OPRs function in α-linolenic acid metabolism and oxylipin biosynthesis (Weber, 2002), well-studied oxygenated fatty acid derivates known for their function as defense molecules in algae (Wasternack, 2007) and especially in diatoms (Pohnert, 2002). A targeted lipidomics analysis for fatty acids and oxylipins was performed to check if indeed the production of these compounds was increased in the presence of bacterial exudates. Arachidonic acid, a fundamental polyunsaturated fatty acid involved in cell signaling (Piomelli, 1993) and inflammation (Calder, 2011) and also synthesized by diatoms (Dunstan et al., 1993), was the only detectable oxylipin in our metabolomics analysis. This is possibly because it is one of the most abundant and important precursor for a range of oxylipins (Pohnert and Boland, 2002; Rettner et al., 2018). The concentration of released arachidonic acid was significantly higher in both SIP + M and SIP + R when compared to induced axenic conditions (SIP) and also in the presence of *Roseovarius* exudates without SIP^+^ (R) compared to the axenic control (C) (Figure 5B). We further investigated oxylipins that were also measured by Rettner et al. (2018), but could find no upregulation in any treatment. Oxylipins were so far predominantly detected from lysed or damaged diatom cells (Pohnert and Boland, 2002), but recently it was suggested that these compounds could have a role in diatom resistance against algicidal bacteria (Meyer et al., 2018) and our study expands this concept even further.

### Comparative Metabolomics Reflects the Different Effects of *Roseovarius* sp. and *Maribacter* sp. Exudates

The medium of *S. robusta* cultures in different treatments was used for metabolomic analysis to gain insights into chemical responses of the induced *S. robusta* cells exposed to bacterial exudates. A principal component analysis (PCA) of both bacterial exudates treatments and an axenic control in presence of SIP^+^ (SIP + M, SIP + R, SIP) shows that the exometabolome of *S. robusta* changes under the influence of bacterial exudates, but the separation of the groups is small (Supplementary Figure S3). We therefore decided to analyze the *Roseovarius* and *Maribacter* datasets separately to highlight potential differences between the two bacterial treatments.

**FIGURE 7:**
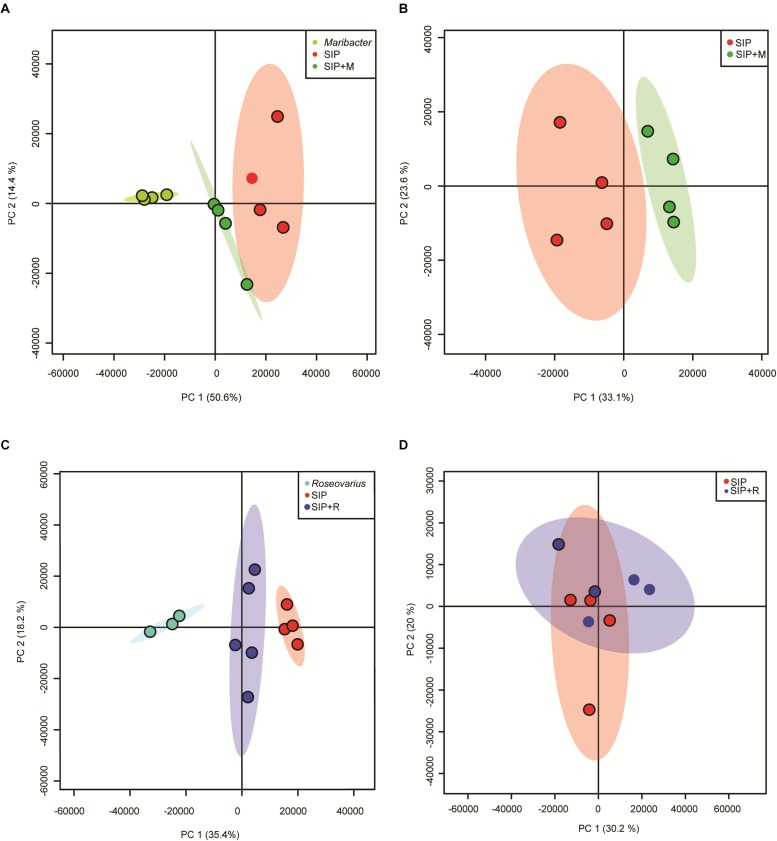
PCA scores plot of exometabolome samples of SIP^+^-induced cultures and bacteria exudates. **(A)** PCA of SIP axenic control, induced cultures + *Maribacter* sp. exudates and *Maribacter* sp. exudates alone. **(B)** PCA of SIP axenic control and induced cultures + *Maribacter* sp. exudates with subtraction of features from *Maribacter* sp. exudates alone. **(C)** PCA of SIP axenic control, induced cultures + *Roseovarius* sp. exudates and *Roseovarius* sp. exudates. **(D)** PCA of SIP axenic control and induced cultures + *Roseovarius* sp. exudates with subtraction of features from *Roseovarius* sp. exudates.

SIP + M and SIP + R samples clearly clustered separately from *S. robusta* axenic samples (SIP) and from bacterial exudates alone (Figures 7A,C), confirming that both bacterial exudates influenced the *S. robusta* exometabolome. To check if these differences were due to presence of molecules from bacterial exudates or in fact caused by *S. robusta* exometabolites, features found in exudates of the bacteria were removed from the feature list of SIP + M and SIP + R. The PCA plots show a clear separation of *Maribacter* sp. exudates-treated induced cultures (SIP + M) from the induced axenic controls (SIP) (Figure 7B), while cultures treated with *Roseovarius* sp. exudates (SIP + R) are largely overlapping with induced axenic cultures (Figure 7D). When we compared the metabolome of non-induced cultures in presence of bacterial exudates (R and M) to the axenic non-induced controls (C), both *Roseovarius*- (R) and *Maribacter* exudates-treated cultures (M) are overlapping with the controls (Supplementary Figure S4). These results corroborate the outcome of our physiological and transcriptomic analysis, with *Maribacter* sp. having a stronger effect on the sexual reproduction and the metabolism of sexually induced MT^–^ *S. robusta* cells.

**FIGURE 8:**
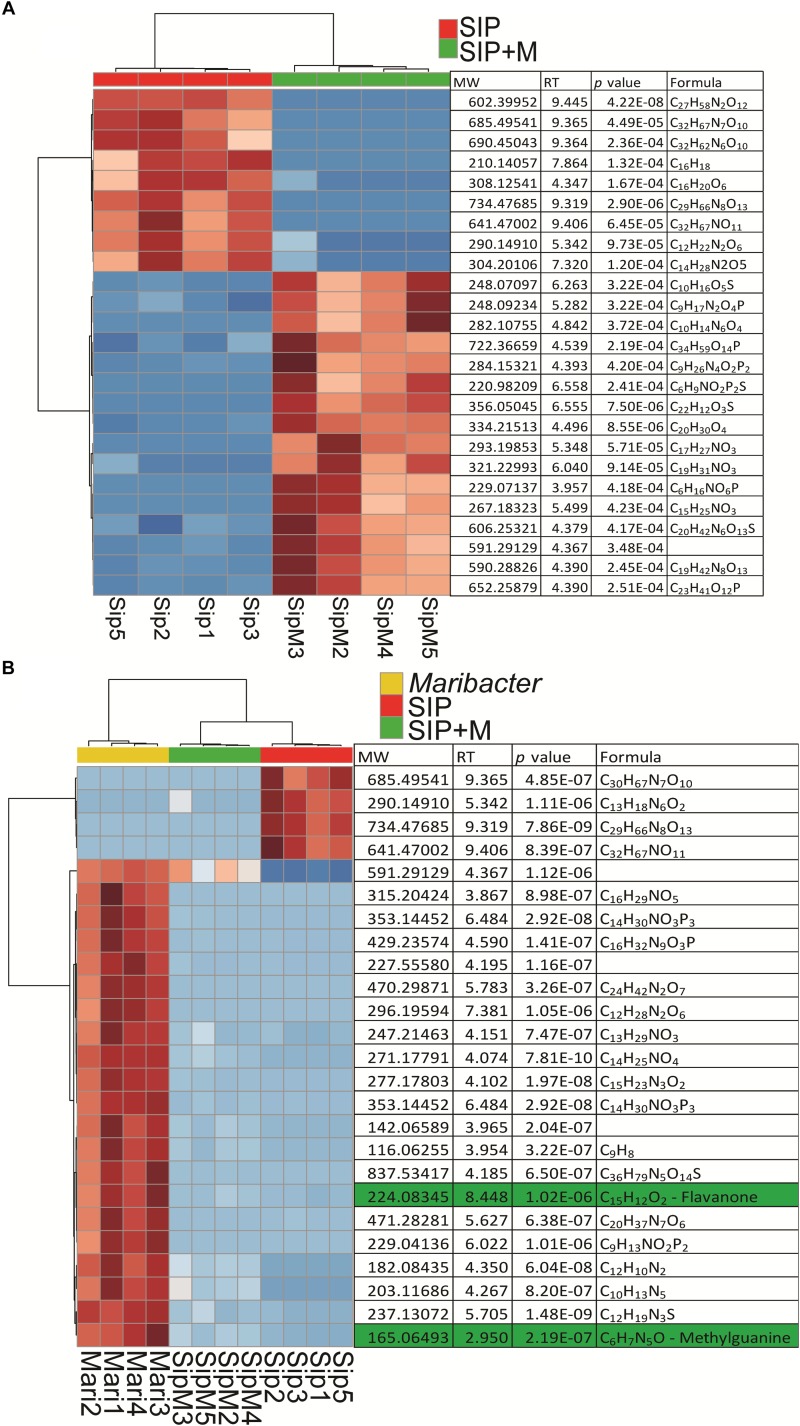
Heatmaps of up- and downregulated *S. robusta* exometabolites: **(A)** in presence or absence of *Maribacter* exudates after subtraction of *Maribacter* sp. features and **(B)** in presence or absence of *Maribacter* exudates and up- and downregulated exometabolites from *Maribacter* sp. For two way comparisons, significance was evaluated with a *t*-test (α = 0.05), hierarchical clustering is based on Euclidean distances and using Ward’s method. For multiple comparisons, significance was evaluated with a one-way ANOVA (adjusted *p*-value after Fisher LSD *post hoc* test = 0.05), hierarchical clustering was based on Euclidean distances and using Ward’s method. Red is for upregulated metabolites and blue is for downregulated metabolites.

We therefore used a comparative metabolomics approach to investigate the exudates of *Maribacter* sp. and the exometabolome of induced (SIP^+^-treated) *S. robusta* when exposed to *Maribacter* sp. exudates (SIP + M) to search for putative signaling molecules. We performed a one-way ANOVA (FDR cutoff = 0.05, Fisher LSD *post hoc* analysis) to select for significant features and chose the top 25 among them (ranked by adjusted *p-*value). Although most of the molecules were identified as unknown, retention times allowed a classification based on their polarity (Figure 8A). Most of the upregulated compounds in SIP + M treatment ranged from mid-polar to non-polar, eluting between 4.5 and 9 min (from 45% of acetonitrile to 100% of acetonitrile solvent composition), while many of the upregulated molecules in SIP medium were non-polar, eluting after 9 min. When we included molecules from bacterial exudates in the analysis (Figure 8B), several compounds released in the medium by *Maribacter* sp. showed a high chromatographic peak intensity that was significantly decreased in SIP + M treatment, suggesting the potential involvement of a signaling mechanism in which the bacterial compound could be degraded by the diatom. In particular, two compounds, eluting at 2.95 min (MW = 165.06493 Da, putative chemical molecular formula C_6_H_7_N_5_O) and at 8.45 min (MW = 224.08345 Da, putative molecular formula C_15_H_12_O_2_) had high peak intensities in *Maribacter* sp. exudates (peak intensity = 10^6^), while their intensities were, respectively, three and one order of magnitude lower in SIP + M treatments. Moreover, these compounds were not present in high amounts in *Roseovarius* sp. exudates (Supplementary Figure S5). After obtaining a fragmentation tree from our MS/MS data and comparing it to public and in-house libraries, we got putative structure for the two compounds: the first one was annotated as a presumed methylguanine, a methyl derivative of the nucleobase guanine, while the second one was annotated as a small weight flavanone (Figure 6B). Methylguanines are naturally occurring modified purines from tRNA in humans (De Bont and van Larebeke, 2004) but they are not known to be produced by bacteria as exometabolites. Flavanones are a type of flavonoids that often occur in plants and have several functions, from antioxidants to antimicrobial (Cushnie and Lamb, 2005), and were also found in a *Pseudovibrio* sp. (Crowley et al., 2014). However, flavanone production by other marine bacteria as well as a function in inter-kingdom crosstalk has not yet been described. Further metabolomics experiments using larger volumes of bacterial exudates and *S. robusta* are needed to better elucidate the nature of these compounds. Moreover, fractionation-guided bioassays may explain their biological function.

## Conclusion

Bacteria associated to *S. robusta* are able to modulate diproline concentrations in the medium and two of them (*Roseovarius* sp. and *Maribacter* sp.) have an opposite effect on the sexual efficiency of *S. robusta*, with *Maribacter* sp. reducing mating efficiency and *Roseovarius* sp. slightly improving it (Cirri et al., 2018). This effect is observed also when *S. robusta* cultures are treated with exudates from this two bacteria (Cirri et al., 2018). Following these findings, we now provide the first insights into the bacterial exudates effect on sexual reproduction of *S. robusta* on a molecular level using a combination of physiological, metabolomic, and transcriptomic approaches. With the integration of different data types, we were able to conclude that both bacterial exudates do not directly interfere with cell cycle arrest and expression of genes related to sexual reproduction of *S. robusta*. Rather, *Roseovarius* sp. exudates cause an increase of proline biosynthetic activity, whereas *Maribacter* sp. exudates influence amino acid and LHC biosynthetic processes. We hypothesize that these two distinct responses lead to opposite effects on production of the attraction pheromone diproline released by *S. robusta*. Moreover, both bacterial exudates are triggering an oxidative stress response in the diatom, which is involving fatty acid metabolism and oxylipin production. It is important to highlight that in addition to the annotated DE genes discussed here, several highly up- and downregulated genes in all treatments were lacking a functional annotation. Better annotations will provide future studies with more knowledge to unravel the influence of bacteria on diatom sexuality and metabolic regulation. These results will pave the way to a better understanding of diatoms life cycle regulation in natural environments and more generally of the importance of inter-kingdom signaling for diatom reproduction and survival.

## Data Availability

The datasets generated for this study can be found in the Gene Expression Omnibus, https://www.ncbi.nlm.nih.gov/geo/query/acc.cgi?acc=GSE131727.

## Author Contributions

EC, SDD, GB, and MW performed the experiments and analyzed the data. EC and SDD analyzed the transcriptomics data. EC analyzed the metabolomics data. GB analyzed the flow cytometry data. CO-C and KV performed the gene model prediction. MW analyzed the oxylipins concentration. EC, SDD, WV, and GP conceived the experiments and the experimental setup. EC, SDD, WV, and GP wrote the manuscript. All authors reviewed the manuscript and the results.

## Conflict of Interest Statement

The authors declare that the research was conducted in the absence of any commercial or financial relationships that could be construed as a potential conflict of interest.

## Footnotes

1 http://bccm.belspo.be

2 http://www.R-project.org/

3 http://hannonlab.cshl.edu/fastx_toolkit/index.html

4 http://revigo.irb.hr/

5 http://bioinfogp.cnb.csic.es/tools/venny/index.html

6 www.graphpad.com
