# Supplementary material for: Associated Bacteria Affect Sexual Reproduction by Altering Gene Expression and Metabolic Processes in a Biofilm Inhabiting Diatom
Source: Front Microbiol. 2019 Aug 2;10:1790. doi: 10.3389/fmicb.2019.01790 (PMC6688387; doi:10.3389/fmicb.2019.01790)
Supplement: Supplementary file 1 [file Table_1.docx]

Supplementary material

# **Supplementary figures and tables**


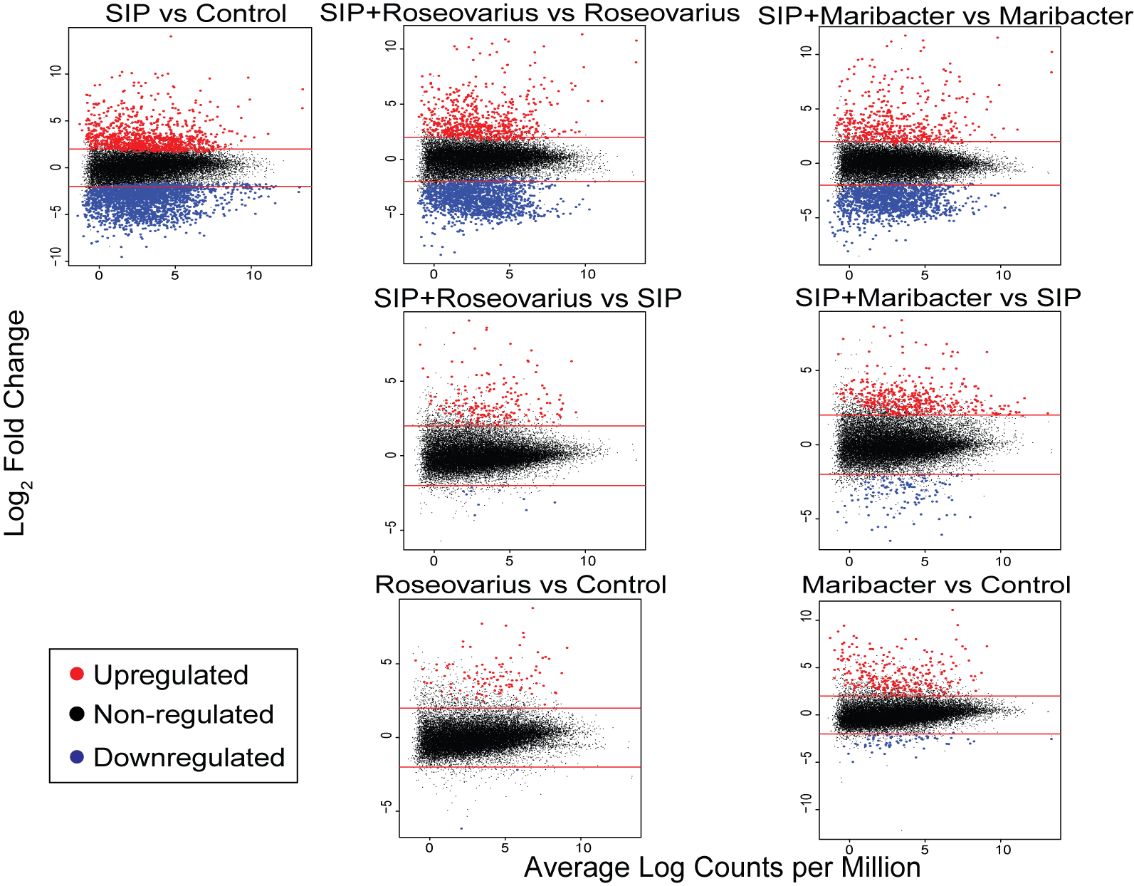


**Figure S1: Mean-difference plot of log_2_ fold change versus the average log2-count per millions in different comparisons of different treatments.**


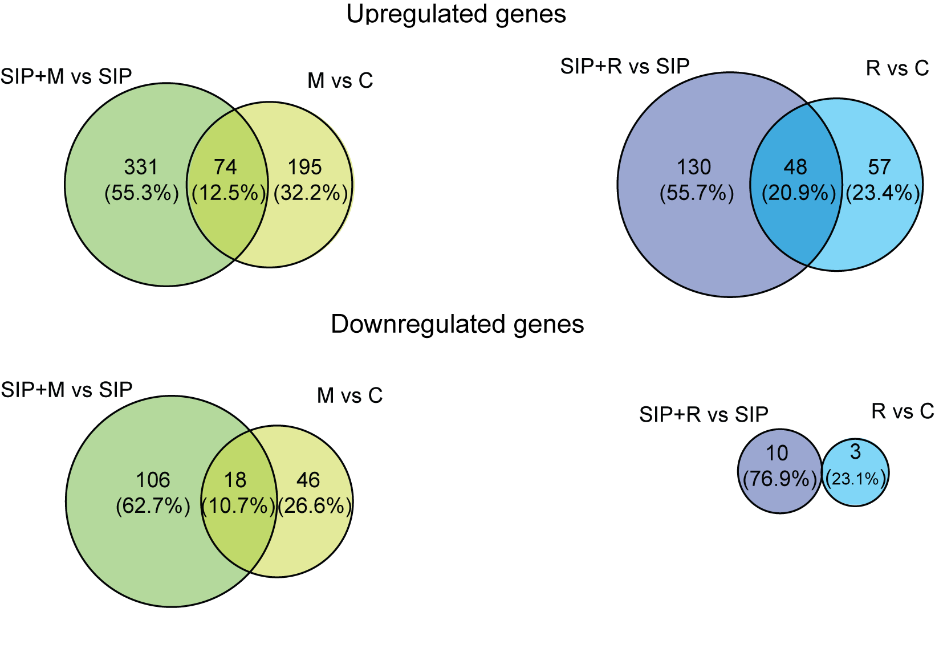


d

b

c

a

**Figure S2**: **Venn diagrams of up- (a and b) and downregulated (b and c) *S. robusta* genes under bacterial influence.** a) and c) refers to SIP+ induced versus non-induced *S. robusta* in presence of *Maribacter* sp. medium, b) and d) to SIP+ induced versus non-induced *S. robusta* in presence of *Roseovarius* sp. medium.


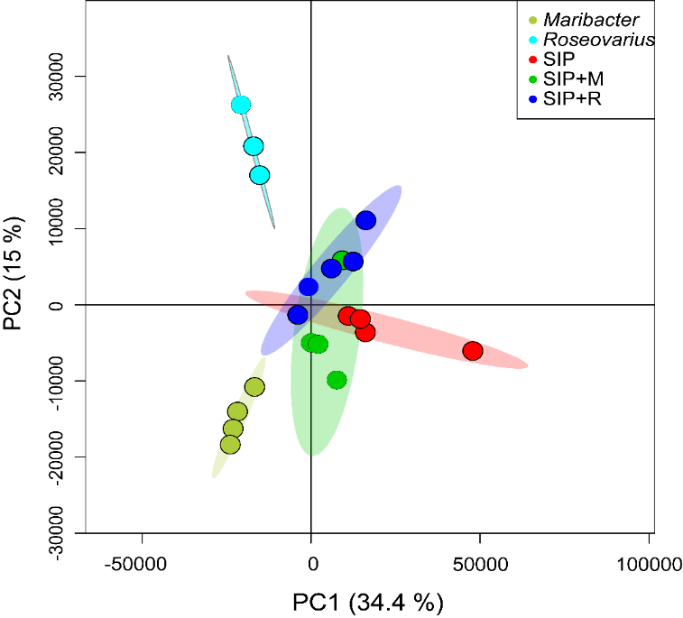


**Figure S3: PCA of exometabolome samples of SIP^+^-induced cultures and bacteria exudates.** Each dot represent a sample for each treatment. Red dots are axenic induced cultures, green dots are induced cultures treated with *Maribacter* sp. exudates, blue dots are induced cultures treated with *Roseovarius* sp. exudates, light blue dots are exudates from *Roseovarius* sp. cultures and light green dots are exudates from *Maribacter* sp.


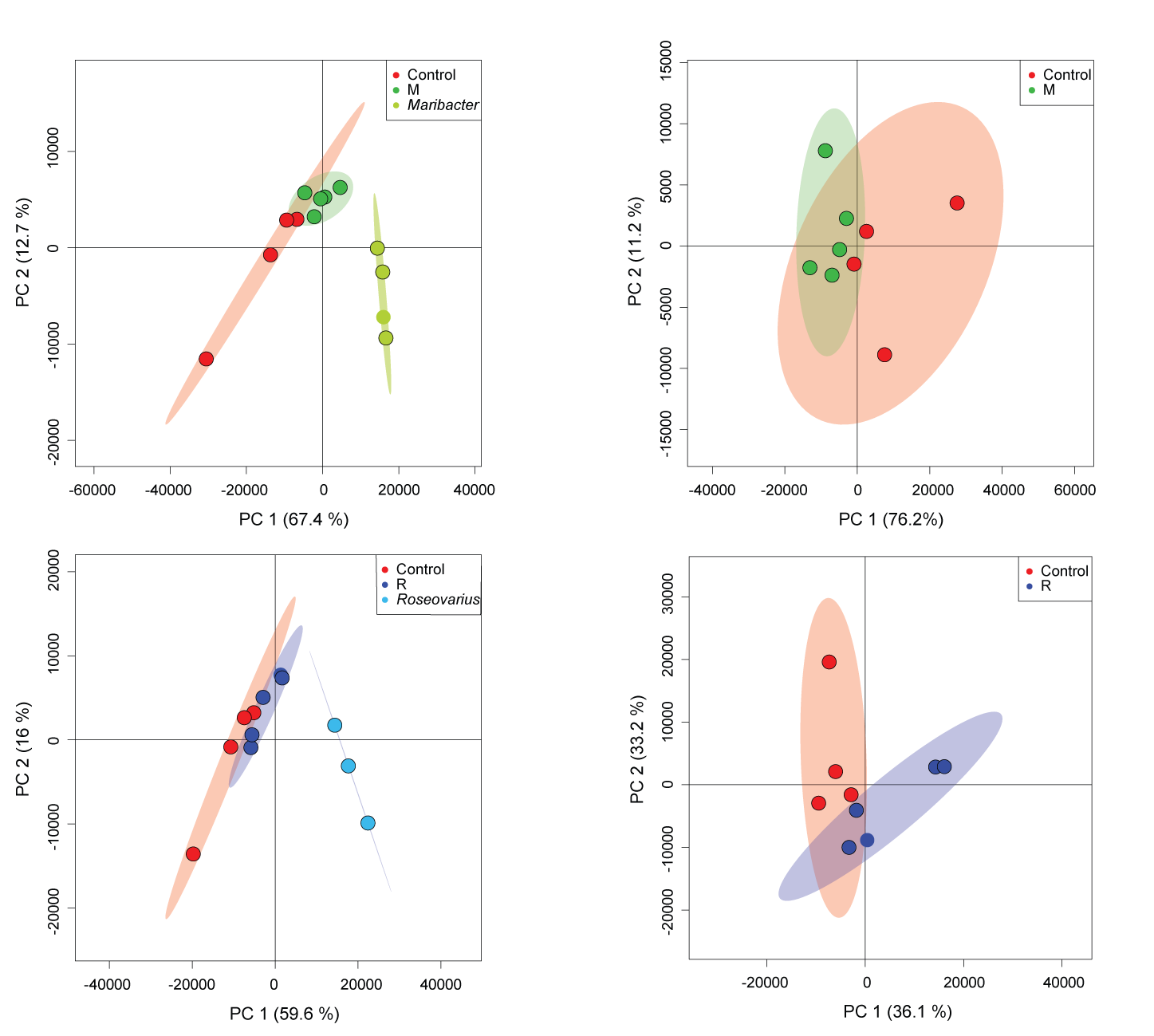


d

c

b

a

**Figure S4:** **PCA of exometabolome samples of non-induced cultures and bacteria exudates.** a) PCA of axenic control, axenic control + *Maribacter* sp. exudates (M) treatment and *Maribacter* sp. exudates, b) PCA of axenic control and axenic control + *Maribacter* sp. exudates (M) with subtraction of features from *Maribacter* sp. exudates, c) PCA of axenic control, axenic control + *Roseovarius* sp. exudates (R) and *Roseovarius* sp. exudates, d) PCA of axenic control and axenic control + *Roseovarius* sp. exudates (R) with subtraction of features from *Roseovarius* sp. exudates.


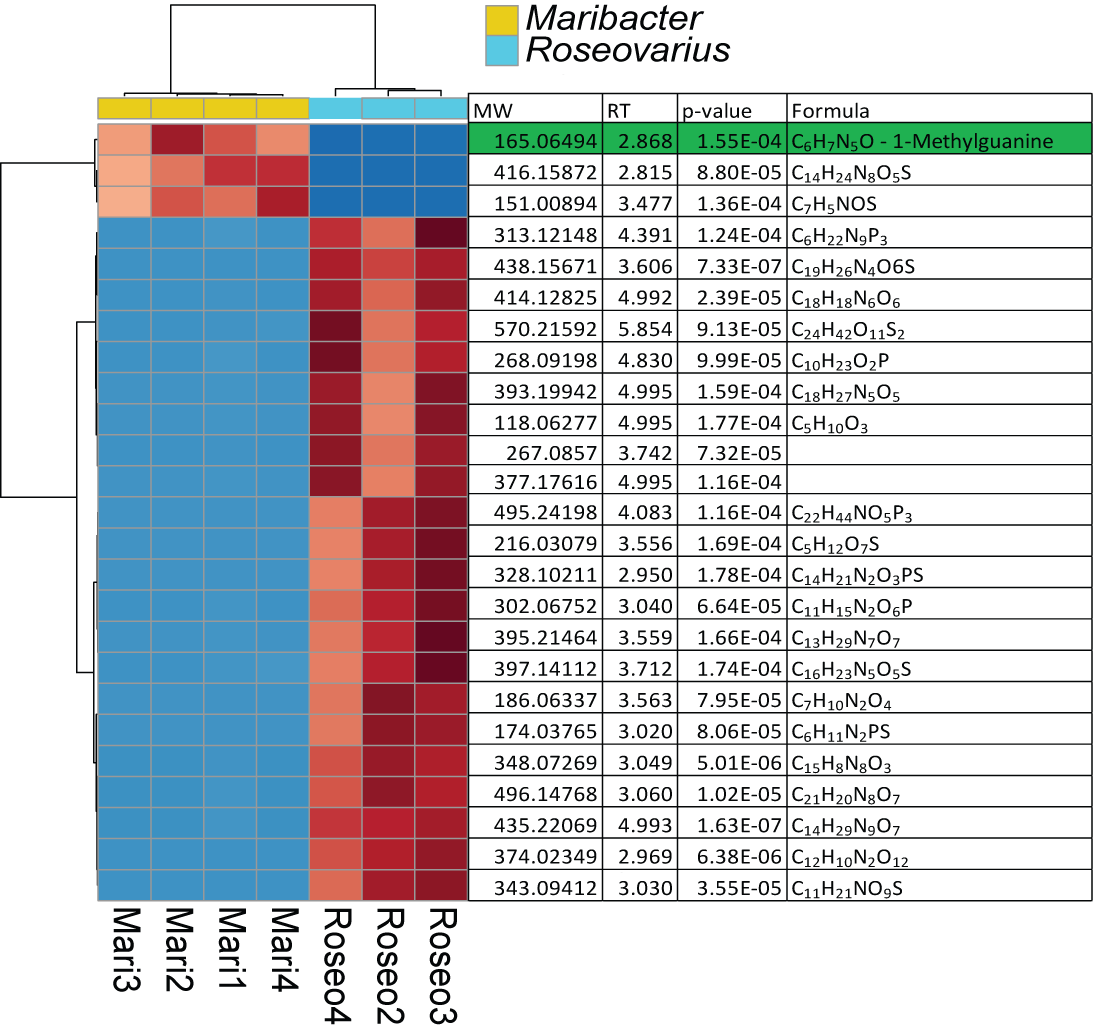


**Figure S5: Heatmaps of up- and downregulated exometabolites from *Maribacter* sp. exudates and *Roseovarius* sp. exudates.** Significance evaluated with a t-Test (α=0.05), hierarchical clustering is based on Euclidean distances and using Ward’s method. Red is for upregulated metabolites, blue is for downregulated metabolites.

**Table S1: GO enrichment results of genes upregulated in all SIP^+^-induced cultures compared to non-induced controls (SIP vs C, SIP+M vs M, SIP+R vs R).**

**Table S2: GO enrichment results of genes downregulated in all SIP^+^-induced cultures compared to non-induced controls (SIP vs C, SIP+M vs M, SIP+R vs R).**

**Table S3: Upregulated genes in presence of *Roseovarius* sp. exudates in SIP^+^-induced cultures (SIP+R vs SIP)**

**Table S4: GO enrichment of genes upregulated in presence of *Roseovarius* sp. exudates in SIP^+^-induced cultures (SIP+R vs SIP)**

**Table S5: Downregulated genes in presence of *Roseovarius* sp. exudates in SIP^+^-induced cultures (SIP+R vs SIP)**

**Table S6: GO enrichment of genes downregulated in presence of *Roseovarius* sp. exudates in SIP^+^-induced cultures (SIP+R vs SIP)**

**Table S7: Upregulated genes in presence of *Maribacter* sp. exudates in SIP^+^-induced cultures (SIP+M vs SIP).**

**Table S8: Downregulated genes in presence of *Maribacter* sp. exudates in SIP^+^-induced cultures (SIP+M vs SIP).**

**Table S9: GO enrichment of genes downregulated in presence of *Maribacter* sp. exudates both in presence and in absence of SIP^+^ (SIP+M vs SIP, M vs C).**

**Table S10: Upregulated genes in presence of both bacterial exudates in SIP^+^-induced cultures (SIP+M vs SIP and SIP+R vs SIP).**

**Table S11: GO enrichment of genes upregulated in presence of both bacteria exudates in SIP^+^-induced cultures (SIP+M vs SIP and SIP+R vs SIP).**
